# Impact of linkage disequilibrium heterogeneity along the genome on genomic prediction and heritability estimation

**DOI:** 10.1186/s12711-022-00737-3

**Published:** 2022-06-27

**Authors:** Duanyang Ren, Xiaodian Cai, Qing Lin, Haoqiang Ye, Jinyan Teng, Jiaqi Li, Xiangdong Ding, Zhe Zhang

**Affiliations:** 1 Guangdong Provincial Key Lab of Agro-Animal Genomics and Molecular Breeding, College of Animal Science, South China Agricultural University, Guangzhou, 510642 China; 2 Key Laboratory of Animal Genetics and Breeding of the Ministry of Agriculture and Rural Affairs, National Engineering Laboratory for Animal Breeding, College of Animal Science and Technology, China Agricultural University, Beijing, 100193 China

## Abstract

**Background:**

Compared to medium-density single nucleotide polymorphism (SNP) data, high-density SNP data contain abundant genetic variants and provide more information for the genetic evaluation of livestock, but it has been shown that they do not confer any advantage for genomic prediction and heritability estimation. One possible reason is the uneven distribution of the linkage disequilibrium (LD) along the genome, i.e., LD heterogeneity among regions. The aim of this study was to effectively use genome-wide SNP data for genomic prediction and heritability estimation by using models that control LD heterogeneity among regions.

**Methods:**

The LD-adjusted kinship (LDAK) and LD-stratified multicomponent (LDS) models were used to control LD heterogeneity among regions and were compared with the classical model that has no such control. Simulated and real traits of 2000 dairy cattle individuals with imputed high-density (770K) SNP data were used. Five types of phenotypes were simulated, which were controlled by very strongly, strongly, moderately, weakly and very weakly tagged causal variants, respectively. The performances of the models with high- and medium-density (50K) panels were compared to verify that the models that controlled LD heterogeneity among regions were more effective with high-density data.

**Results:**

Compared to the medium-density panel, the use of the high-density panel did not improve and even decreased prediction accuracies and heritability estimates from the classical model for both simulated and real traits. Compared to the classical model, LDS effectively improved the accuracy of genomic predictions and unbiasedness of heritability estimates, regardless of the genetic architecture of the trait. LDAK applies only to traits that are mainly controlled by weakly tagged causal variants, but is still less effective than LDS for this type of trait. Compared with the classical model, LDS improved prediction accuracy by about 13% for simulated phenotypes and by 0.3 to ~ 10.7% for real traits with the high-density panel, and by ~ 1% for simulated phenotypes and by − 0.1 to ~ 6.9% for real traits with the medium-density panel.

**Conclusions:**

Grouping SNPs based on regional LD to construct the LD-stratified multicomponent model can effectively eliminate the adverse effects of LD heterogeneity among regions, and greatly improve the efficiency of high-density SNP data for genomic prediction and heritability estimation.

**Supplementary Information:**

The online version contains supplementary material available at 10.1186/s12711-022-00737-3.

## Background

Since genomic prediction was proposed [1], the methods for genomic prediction have undergone considerable optimizations to adapt to traits with different genetic architectures and to populations with different genetic backgrounds. Nevertheless, with the development of quantitative genetics and genome sequencing technologies, there is still room for further optimization of genomic prediction methods. Currently, many statistical learning methods such as the Bayesian methods [2, 3] and machine learning methods [4, 5] have been applied to genomic prediction. Most of these methods focus on improving the estimates of marker effects to optimize the prediction accuracy of the model, but little attention is paid to some other key factors that affect genomic prediction, such as linkage disequilibrium (LD) between single nucleotide polymorphisms (SNPs).

In general, LD information between markers is used to pre-select markers or construct LD-based haplotypes, but these processes have little impact on the accuracy of genomic prediction [6–8]. However, another factor that seems to have a greater impact on genomic prediction is the uneven distribution of LD along the genome, i.e. the LD heterogeneity among regions. Contributions of genetic variance are overestimated for causal variants in regions of high LD and are underestimated in regions of low LD. Several methods have been developed to eliminate the adverse effects of LD heterogeneity among regions on the unbiasedness of heritability estimates, among which LD-adjusted kinship (LDAK) [9] and LD-stratified multicomponent restricted maximum likelihood estimation (GREML-LDS) [10, 11] are widely used. LDAK constructs an LD-weighted genomic relationship matrix (GRM) by assigning small weights to SNPs in high LD regions and large weights to SNPs in low LD regions [9]. GREML-LDS groups SNPs by regional [10] or individual [11] SNP LD score and constructs the GRM with SNPs in each group separately. Previous studies have shown that LDAK and GREML-LDS can better ensure the unbiasedness of heritability estimates for human complex and disease traits [11, 12]. Other studies have found that controlling LD heterogeneity among regions can also improve the unbiasedness of heritability estimation [13] and the accuracy of genomic prediction [14] of the marker effect model. However, there are few studies on genomic selection methods for controlling LD heterogeneity among regions in livestock. Although a previously proposed LD-corrected GRM achieved good results in heritability estimation and genomic prediction, this method seems to be suitable only for low-density SNP panels [15].

Whole-genome sequence (WGS) data and high-density SNP data have been used in animal genetic evaluation [16, 17]. Compared with SNP chip data (i.e., medium-density SNP data), high-density SNP data provide more information, but how to use this information effectively remains a challenge. Recent studies have found that, compared with medium-density SNP data, the use of high-density SNP data has no advantage or even results in a decline in genomic prediction [7, 18, 19]. The GRM constructed in the classical genomic prediction model can accurately capture the relationship between individuals using medium-density markers. However, with the increase in marker density, this method to construct GRM does not seem to be able to explain the relationship between individuals more accurately. Therefore, classical genomic prediction methods cannot make full use of the information provided by high-density SNP data and need to be further optimized. Since the LD between adjacent SNPs in high-density panels is stronger than that in medium-density panels, an important reason for the unsatisfactory genomic prediction results obtained with high-density SNP data may be that they are more affected by LD heterogeneity among regions.

It is generally assumed that the greater is the heritability (genetic variance) captured by the model, the higher is the prediction accuracy of the model. However, studies have shown that the variation of the estimates of heritability is not consistent with the variation of genomic prediction accuracy, i.e. the genomic prediction accuracy does not necessarily increase or it even decreases as the estimates of heritability increase [18]. This makes the results of many methods difficult to understand [20–22]. Therefore, it is necessary to further study the relationship between the estimates of heritability and genomic prediction accuracy.

In this study, we compared a series of models that control LD heterogeneity among regions with the classical model [23] to: (1) find effective models to eliminate the adverse effects of LD heterogeneity among regions and to optimize genomic prediction and heritability estimation, (2) determine whether the models that control LD heterogeneity among regions are more effective with high-density SNP data, and (3) determine why genomic prediction accuracy and estimates of heritability vary inconsistently, and find a unified indicator to measure the model's performance in genomic prediction and SNP-heritability estimation.

## Methods

### Population and genotypes

This study used a German dairy cattle population of 2000 bulls from Vereinigte Informationssysteme Tierhaltung Wirtschaftlicher Verein, which has previously been described in [24]. All individuals were genotyped with the Illumina Bovine SNP50 Beadchip (~ 54,000 SNPs). One of our previous studies used Beagle 4.0 [25] to impute the 2000 bulls genotyped with 54K SNP chip data to 770K SNP data [16], which were also used in this study. Genotype imputation was based on a multi-breed reference population that included 1577 cattle from the fifth run of the 1000 Bull Genomes project [26], of which 474 were Holstein breed. WGS data are available for all the individuals in this reference population. In our imputation process, first we extracted the corresponding loci on the Illumina BovineHD (~ 770,000 SNPs) from the WGS data to construct a 770K high-density reference panel, and then used this high-density reference panel to impute 54K chip data to 770K SNP data. The consistency rate of the genotype imputation was 0.99 through masked analysis. The masked analysis was implemented by randomly masking the genotypes of 100 loci in the 54K chip and calculating the consistency between the imputed and the true genotypes (repeated 20 times). After genotype imputation, SNPs with a minor allele frequency (MAF) lower than 0.01, a genotype call rate lower than 0.9, or that deviated from Hardy–Weinberg equilibrium (p < 10^–6^) were removed; after filtering, 336,977 SNPs (referred to as the 300K SNP panel hereafter) remained for further analyses.

### Phenotypes

#### Simulated phenotypes

We established a series of simulation studies to explore the effects of LD heterogeneity among regions on genomic prediction and heritability estimation. The real dairy cattle genotypic data were used as a base for the simulations. Heritability and number of causal variants were fixed at 0.8 and 100, respectively. Using ${\sum }_{k=1}^{M}{r}_{jk}^{2}$ as a measure of the tagging of SNP $j$ [27], where ${r}_{jk}^{2}$ denotes the squared correlation between SNPs $j$ and $k$, $M$ the number of SNPs in the 10-Mb region centered on SNP $j$, we defined those in the bottom 40 and 20% of values for this sum as weakly and very weakly tagged SNPs, respectively, and those in the top 40 and 20% as strongly and very strongly tagged SNPs. We selected the causal variants from weakly, very weakly, strongly, and very strongly tagged SNPs, respectively. At the same time, SNPs were randomly selected throughout the genome to obtain causal variants with average tagging levels. Thus, five scenarios of phenotypes with causal variants of different tagging levels (very weak, weak, average, strong, very strong) were produced. The above-method of selection of causal variants refers to an earlier study [9]. The phenotypic variance ${\delta }_{p}^{2}$ was set to 1. The allele substitution effect of the $i$-th causal variant ${a}_{i}$ was calculated as ${a}_{i}={(2{p}_{i}\left(1-{p}_{i}\right))}^{-1/2}{\delta }_{g}/\sqrt{m}$, where ${\delta }_{g}=\sqrt{{\delta }_{p}^{2}\times {h}^{2}}$ represents the genetic standard deviation caused by all causal variants, ${p}_{i}$ is the frequency of a given allele of the $i$-th causal variant, and $m$ is the total number of causal variants. All GRM in this study were constructed based on the method proposed by Yang et al. [23]. This method to construct GRM assumes that all the causal variants contribute equally to heritability, i.e., all ${h}_{i}^{2}=2{p}_{i}(1-{p}_{i}){a}_{i}^{2}$ are equal. Our simulation of the allele substitution effect was designed to satisfy this assumption. This simulation method avoids the bias of heritability estimates caused by inconsistency between the simulated and assumed effect of causal variants during GRM construction. Therefore, only the LD level of the causal variants had an effect on heritability estimation in the simulation study. Environmental effects were drawn from a normal distribution $N(0,\left(1-{h}^{2}\right){\delta }_{p}^{2})$. The simulated phenotype of an individual was calculated as the sum of the effects of its causal variant and an environmental effect, each scenario was repeated 100 times. Our phenotypic simulation scripts are available at https://github.com/SCAU-AnimalGenetics/LD-heterogeneity/tree/main/simphe.

Previous studies have shown that the genomic prediction accuracy of medium-density panels (20 to 50K) with SNPs evenly distributed throughout the genome is higher than that of high-density SNP panels [18]. In the simulation study, we constructed a medium-density panel (50K) using evenly distributed SNPs and compared the medium- and high-density panels in terms of genomic prediction and heritability estimation. In this part of the study, the causal variants in the medium- and the high-density panels were removed. In the study on real traits, we used the 54K commercial SNP panel (Illumina Bovine SNP50 Beadchip) as the medium-density SNP panel.

#### Real dairy cattle traits

Pedigree-based estimated breeding values (EBV) for milk yield (MY), milk protein yield (PY), milk fat yield (FY), milk protein percentage (PP), milk fat percentage (FP) and somatic cell score (SCS) were available for all the bulls. Additional file 1: Table S1 presents the descriptive statistics of the EBV and their reliabilities and shows that the reliability of the EBV of all the traits is high (mean reliability ranging from 0.942 to 0.973), and that the variation in reliability is small (standard deviation ranging from 0.016 to 0.039), thus there was no obvious heterogeneity in EBV reliability. In this study, we standardized EBV for each trait so that the mean value of EBV was zero and the variance was 1. Since for most individuals in this population, only their own EBV are available and not those of their parents, we used ${g}_{i}/{r}_{i}^{2}$ to calculate deregressed EBV of each individual, where ${g}_{i}$ represents the EBV of the $i$-th individual and ${r}_{i}^{2}$ represents the reliability of ${g}_{i}$ [28]. Deregressed EBV were used as phenotypes in this study.

### Models for genomic prediction and heritability estimation

#### Genome-wide complex trait analysis (GCTA) model

The GCTA model [23] was used as the benchmark method in this study. It includes a single random genetic effect and is as follows:

1 $$\mathbf{y}={\varvec{\upmu}}+\mathbf{Z}\mathbf{g}+\mathbf{e},$$

where $\mathbf{y}$ is the vector of phenotypes, ${\varvec{\upmu}}$ is a vector of the overall mean, $\mathbf{g}$ is a vector of individual genetic values captured by all SNPs in the panel, $\mathbf{Z}$ is the design matrix of genetic values, and $\mathbf{e}$ is a vector of residuals. The random genetic and residual values are assumed to be independent normally distributed values: $\mathbf{g}\sim N(\boldsymbol{0},\mathbf{G}{\upsigma }_{\mathrm{g}}^{2})$ and $\mathbf{e}\sim N(\boldsymbol{0},\mathbf{I}{\upsigma }_{\mathrm{e}}^{2})$, where ${\upsigma }_{\mathrm{g}}^{2}$ and ${\upsigma }_{\mathrm{e}}^{2}$ are the additive genetic variance and residual variance, respectively.

The additive $\mathbf{G}$ matrix, also known as the genomic relationship matrix (GRM), was constructed using all the SNPs in the panel:

2 $${\mathbf{G}\mathbf{R}\mathbf{M}}_{\mathrm{GCTA}}=\frac{\mathbf{X}{\mathbf{X}}^{\prime}}{N},$$

where matrix $\mathbf{X}$ has the general term ${x}_{ij}=({m}_{ij}-2{p}_{j})/\sqrt{2{p}_{j}(1-{p}_{j})}$, with ${p}_{j}$ being the frequency of a given allele at SNP $j$, ${m}_{ij}$ is the genotype of the $j$*-*th SNP in the $i$*-*th individual, which is represented by 0, 1 and 2. $N$ is the number of SNPs in the panel.

#### Linkage disequilibrium adjusted kinship (LDAK) model

Due to LD, SNPs will be repeatedly tagged. Using the LDAK software [9], we calculated the level of replicate tagging of SNPs, which represents the real contribution of SNPs to genomic relationships in the GRM that do not control LD heterogeneity among regions. In the GCTA model, the genetic variance of the causal variants in high LD regions is overestimated, while the genetic variance in low LD regions is underestimated [9]. To offset this adverse effect, we used an LD-weighted GRM to replace the GRM in Eq. (2). Such LD weighting eliminates the overestimation of heritability in high LD regions and the underestimation of heritability in low LD regions by giving a small weight to markers in high LD regions and a large weight to markers in low LD regions. We used ${w}_{j}^{*}={w}_{j}\frac{N}{{\sum }_{j}{w}_{j}}$ to represent the LD weight of SNP $j$, for the derivation and calculation of ${w}_{j}$, please refer to Speed et al. [9]. The LD-weighted GRM is constructed as follows:

3 $${\mathbf{G}\mathbf{R}\mathbf{M}}_{\mathrm{LDAK}}=\frac{\mathbf{X}\mathbf{W}{\mathbf{X}}^{\mathbf{^{\prime}}}}{N},$$

where $\mathbf{W}$ is the diagonal matrix with elements ${w}_{j}^{*}$. To generate the LDAK model, the ${\mathbf{G}\mathbf{R}\mathbf{M}}_{\mathrm{GCTA}}$ in Eq. (1) is replaced by the ${\mathbf{G}\mathbf{R}\mathbf{M}}_{\mathrm{LDAK}}$.

#### GREML-LDS model

In addition to constructing the LDAK model, we also used the LD-stratified multicomponent model to offset the influence of LD heterogeneity among regions. In this study, the regional SNP LD score was used to divide SNPs into five equal groups, corresponding to SNPs with very high, high, moderate, low and very low LD levels, respectively. The SNPs in each group were used to construct the respective GRM and then a multi-component model was established [10]:

4 $$\mathbf{y}={\varvec{\upmu}}+\sum_{t}^{T}{\mathbf{Z}}_{t}{\mathbf{g}}_{t}+\mathbf{e},$$

where $\mathbf{y}$, ${\varvec{\upmu}}$ and $\mathbf{e}$ are the same as for Eq. (1). ${\mathbf{g}}_{t}$ is a vector of the genetic values of the individuals captured by the SNPs in the $t$-th group, and ${\mathbf{g}}_{t}\sim N(\boldsymbol{0},{\mathbf{G}}_{t}{\upsigma }_{{\mathbf{g}}_{t}}^{2})$, with ${\upsigma }_{{\mathbf{g}}_{t}}^{2}$ being the additive genetic variance explained by the SNPs in the $t$-th group, and ${\mathbf{G}}_{t}$ the GRM constructed by the SNPs in the $t$-th group. The ${\mathbf{G}}_{t}$ was constructed by Eqs. (2) or (3), respectively, and the corresponding models are called the GCTA-LDS model or LDAK-LDS model, respectively.

In this study, we used the mean LD score to represent the regional LD. The mean LD score was fitted with ~ 100-kb segments using a sliding window approach [10], and the LD score of each SNP is calculated as the sum of the LD ${r}^{2}$ values for the target SNP and all SNPs within the 10-Mb region centered on the target SNP [27]. Our method to calculate the mean LD score is available on https://github.com/SCAU-AnimalGenetics/LD-heterogeneity/tree-save/main/regional-LD. The GCTA, LDAK, GCTA-LDS and LDAK-LDS models used in this study only consider additive genetic effects, so the GCTA-LDS and LDAK-LDS models did not consider the covariance of genetic effects corresponding to different GRM.

### Model assessment

In this study, variance components and genetic values were estimated using the LDAK software [9]. For each simulation repeat, 1800 individuals were randomly selected as the training population and the residual 200 individuals as the validation population. The prediction accuracy is the Pearson’s correlation coefficient of the true genetic values and genomic estimated breeding values (GEBV) of the validation individuals. For real traits, the 10 × tenfold cross-validation was used to evaluate the models, and the genomic prediction accuracy is expressed as Pearson's correlation coefficient of the deregressed EBV and GEBV. For each trait, a one-way analysis of variance was applied to determine whether there were any statistically significant differences in prediction accuracy, heritability estimate and model fit (Akaike Information Criterion, AIC) between the different models; if the null hypothesis was rejected using the significance level of 0.05, the multiple paired *t*-tests were conducted between all models, with *P* values adjusted by Bonferroni correction.

## Results

### Linkage disequilibrium heterogeneity among regions and uneven tagging of SNPs

**Fig. 1:**
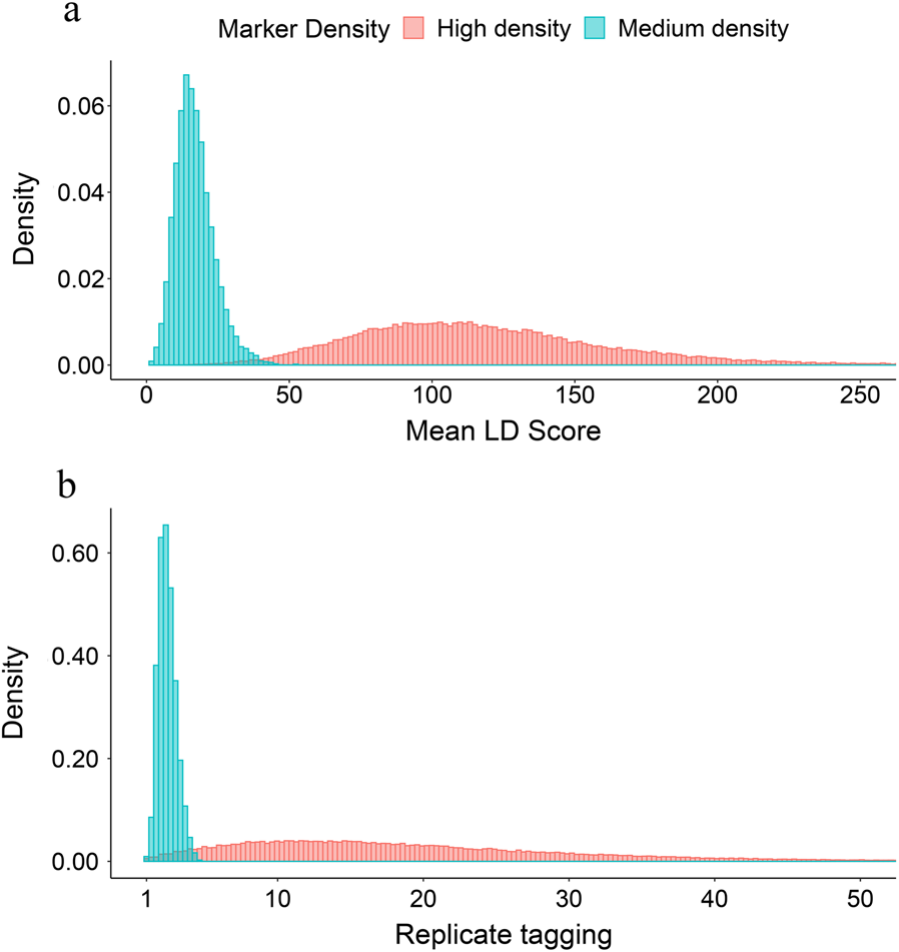
Distribution of mean LD score and level of replicate tagging for SNPs in high- and medium-density panels. **a** The mean LD score is used to represent the regional LD, which was fitted by segment with an average length of 100 kb using a sliding window approach, and the LD score of each SNP is calculated as the sum of the LD ${r}^{2}$ values for the target SNP and all SNPs within the 10-Mb region centered on the target SNP. **b** The level of replicate tagging was calculated using the LDAK software to show that SNPs were repeatedly tagged due to LD

The mean LD score used to represent the regional LD ranged from 3.8 to 347.9 for the high-density panel (Fig. 1a), which indicates large differences in LD among genomic regions. The mean LD score calculated by the medium-density panel was relatively low, ranging from 1.2 to 52.9 (Fig. 1a). The level of replicate tagging of SNPs ranged from 1 to ~ 100.2 for SNPs in the high-density panel and from 1 to ~ 4.9 for SNPs in the medium-density panel (Fig. 1b). After adding LD weights, the level of replicate tagging of SNPs in both the high- and medium-density panels was around 1 (see Additional file 2: Fig. S1). We divided SNPs into five levels according to their mean LD score, corresponding to the genome regions with very high, high, moderate, low and very low LD, respectively (Table 1). The SNPs in each LD level were used to construct the GRM in the GREML-LDS, respectively. Therefore, the GCTA-LDS and LDAK-LDS used in this study have five independent genetic effects. For the high-density panel, the difference in mean LD score of SNPs in adjacent LD levels ranged from 20 to 50, while for the medium-density panel, it was between 3 and 8 (Table 1). Differences in the level of replicate tagging of SNPs between LD levels were significant for the high-density panel and almost null for the medium-density panel (Table 1).

**Table 1:** Genomic regions with different LD levels partitioned by mean LD score

LD levels	High-density panel (300K SNPs)		Medium-density panel (50K SNPs)	
	Mean LD score^a^	Replicate tagging level	Mean LD score	Replicate tagging level
Very high	186.9 (33.0)^b^	26.9 (15.7)^c^	27.1 (4.7)	2.5 (1.0)
High	136.3 (8.0)	21.2 (12.2)	19.7 (1.2)	2.5 (1.0)
Moderate	112.3 (6.1)	18.4 (10.6)	16.1 (0.9)	2.4 (1.0)
Low	91.3 (6.2)	16.3 (9.7)	13.0 (0.9)	2.4 (0.9)
Very low	62.8 (13.5)	14.0 (8.5)	8.7 (2.1)	2.3 (0.9)

^a^^:^Mean LD score was used to represent regional LD

^b^Average mean LD score of SNPs at each LD level (standard deviation)

^c^Average replicate tagging of SNPs at each LD level (standard deviation)

### Performance of the different models in terms of genomic prediction and heritability estimation

The GCTA, LDAK and GREML-LDS models were originally proposed to improve the unbiasedness of heritability estimates. In this study, the performance of each model in terms of genome prediction, heritability estimation and model fit were analyzed simultaneously to fully evaluate the effectiveness of each model in dealing with LD heterogeneity.

**Fig. 2:**
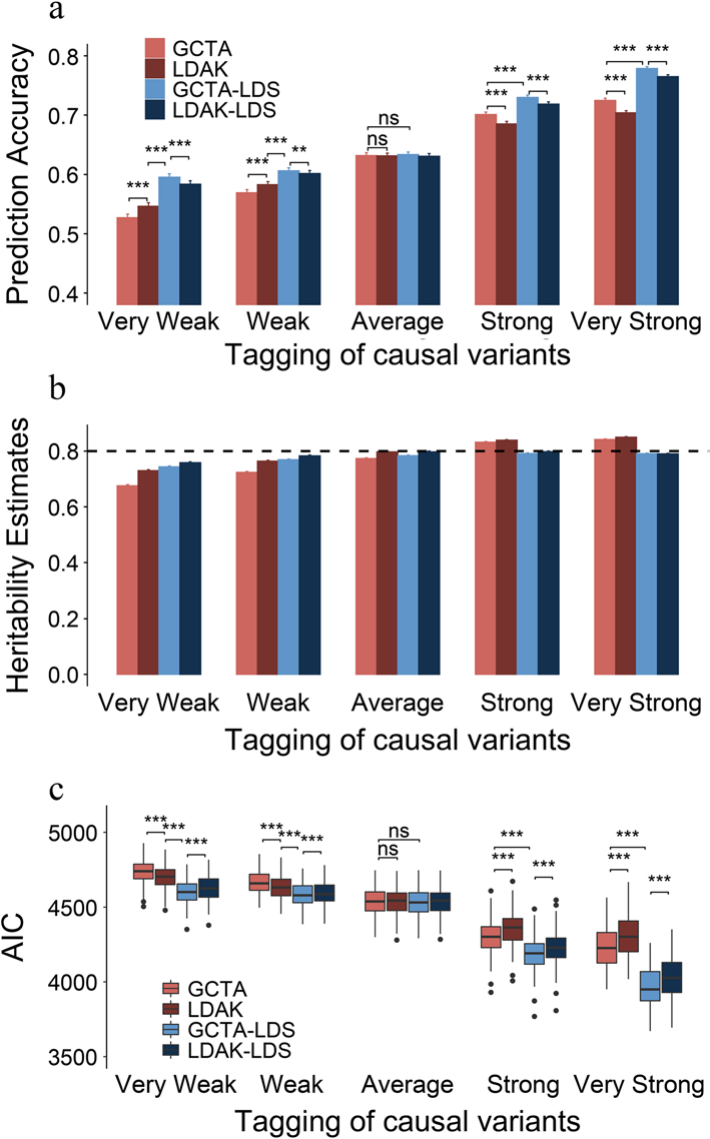
Performance of GCTA, LDAK, GCTA-LDS and LDAK-LDS in terms of genomic prediction (**a**), heritability estimation (**b**), and model fit (**c**) for phenotypes that are controlled by causal variants with different tagging levels. Paired *t*-test was applied to compare the difference between models, with *P* values adjusted by Bonferroni correction. *** indicates significant differences at *P* < 0.001, ** significant differences at 0.001 < *P* < 0.01, * significant differences at 0.01 < *P* < 0.05, and ns indicates no statistically significant difference

Figure 2a represents the genomic prediction accuracy of each model for phenotypes that are controlled by causal variants with different tagging levels. Compared with GCTA, LDAK increased the genomic prediction accuracy of phenotypes that are controlled by weakly (or very weakly) tagged causal variants, but decreased that for phenotypes controlled by strongly (or very strongly) tagged causal variants. Therefore, LDAK is only suitable for genomic prediction of phenotypes that are mainly controlled by weakly tagged causal variants. Compared with GCTA and LDAK, GCTA-LDS and LDAK-LDS can greatly improve the genomic prediction accuracy regardless of whether the phenotype is controlled by weakly or strongly tagged causal variants. For phenotypes that are controlled by evenly distributed causal variants along the genome (causal variants are averagely tagged), the genomic prediction results of the four models were very similar. Figure 2b shows the heritability estimates obtained with the different models. GCTA and LDAK underestimate the heritability of phenotypes that are controlled by weakly (or very weakly) tagged causal variants and overestimate the heritability of phenotypes that are controlled by strongly (or very strongly) tagged causal variants. The heritability estimates of GCTA-LDS and LDAK-LDS were unbiased. The model fit (AIC) was closely related to the accuracy of genomic prediction and the unbiasedness of heritability estimates (Fig. 2a–c), i.e., the higher the genomic prediction accuracy, the better the unbiasedness of heritability estimates, the lower the AIC, and vice versa. In general, GCTA-LDS and LDAK-LDS can effectively eliminate the adverse effects of LD heterogeneity among regions, and improve the unbiasedness of heritability estimation and the accuracy of genomic prediction, regardless of the genetic architecture of the trait. Considering that most economically-important traits in livestock have a heritability estimate lower than 0.5, we also simulated and analyzed traits with a heritability of 0.5 and found that the trend was the same as with a heritability of 0.8 (see Additional file 2: Fig. S2).

### Impact of marker density on genomic prediction and heritability estimation

**Fig. 3:**
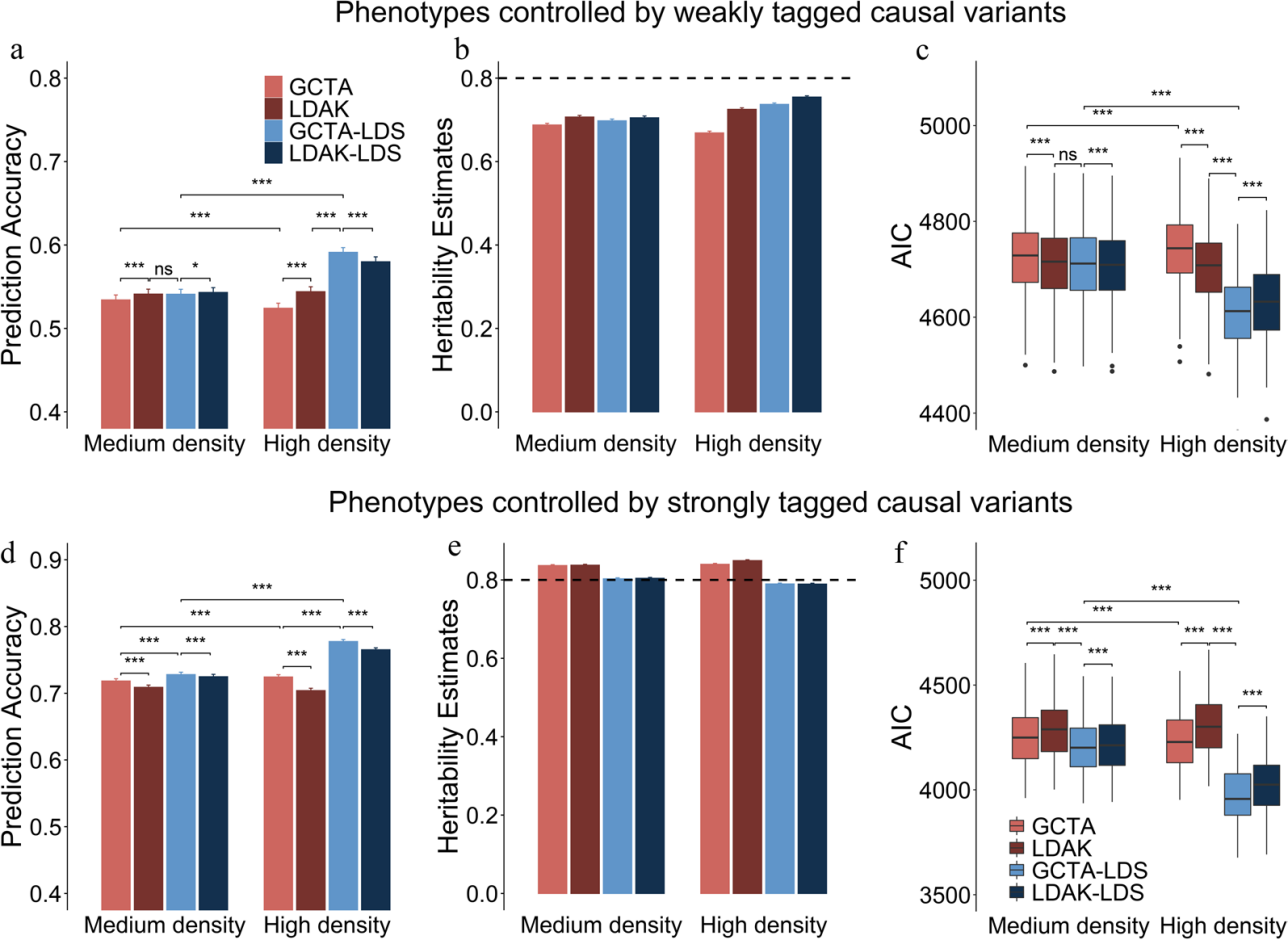
Performance of different models in terms of genomic prediction, heritability estimation, and model fit based on the medium-density (50K SNPs) and high-density (300K SNPs) panels. The three plots in the upper half represent the results for genomic prediction (**a**), heritability estimation (**b**) and model fit (**c**) for phenotypes that are controlled by weakly tagged causal variants. The three plots in the lower half represent the results for genomic prediction (**d**), heritability estimation (**e**) and model fit (**f**) for phenotypes that are controlled by strongly tagged causal variants. Paired *t*-test was applied to compare the difference between methods, with *P* values adjusted by Bonferroni correction. *** indicates significant differences at *P* < 0.001, ** significant differences at 0.001 < *P* < 0.01, * significant differences at 0.01 < *P* < 0.05, and ns indicates no statistically significant difference

Figure 3a–c represents the results of genomic prediction, SNP-based heritability estimation and model fit of each model for phenotypes that are controlled by weakly tagged causal variants. LDAK, GCTA-LDS and LDAK-LDS have advantages over GCTA with both medium- and high-density panels, and the advantages are more obvious with the high-density panel. Compared with GCTA, GCTA-LDS achieved an improvement of ~ 13% in genomic prediction accuracy based on the high-density panel, but of only ~ 1% based on the medium-density panel (Fig. 3a). For GCTA, the genomic prediction accuracy based on the high-density panel was significantly lower than that of the medium-density panel (Fig. 3a). By controlling the LD heterogeneity among regions, GCTA-LDS and LDAK-LDS can effectively use high-density SNP data and significantly improve genomic prediction accuracy compared to medium-density data (Fig. 3a). The heritability estimates of LDAK, GCTA-LDS and LDAK-LDS were higher than those of GCTA but still lower than the true value (Fig. 3b).

Figure 3d–f represents the results of genomic prediction, SNP-based heritability estimation and model fit of each model for phenotypes that are controlled by strongly tagged causal variants. Using GCTA, the high-density panel can improve genomic prediction accuracy compared to the medium-density panel (Fig. 3d). As for the results in Fig. 2, LDAK does not apply to phenotypes that are controlled by strongly tagged causal variants. Compared with GCTA, GCTA-LDS and LDAK-LDS improved genomic prediction accuracy by 7.3% when using the high-density panel and by 1.3% when using the medium-density panel (Fig. 3d). GCTA and LDAK overestimated the heritability, and the heritability estimates of GCTA-LDS and LDAK-LDS were almost unbiased (Fig. 3e). Model fit can be used to reflect the accuracy of genome prediction and the unbiasedness of heritability estimates of the model (Fig. 3c and f).

### Application to real dairy cattle traits

**Fig. 4:**
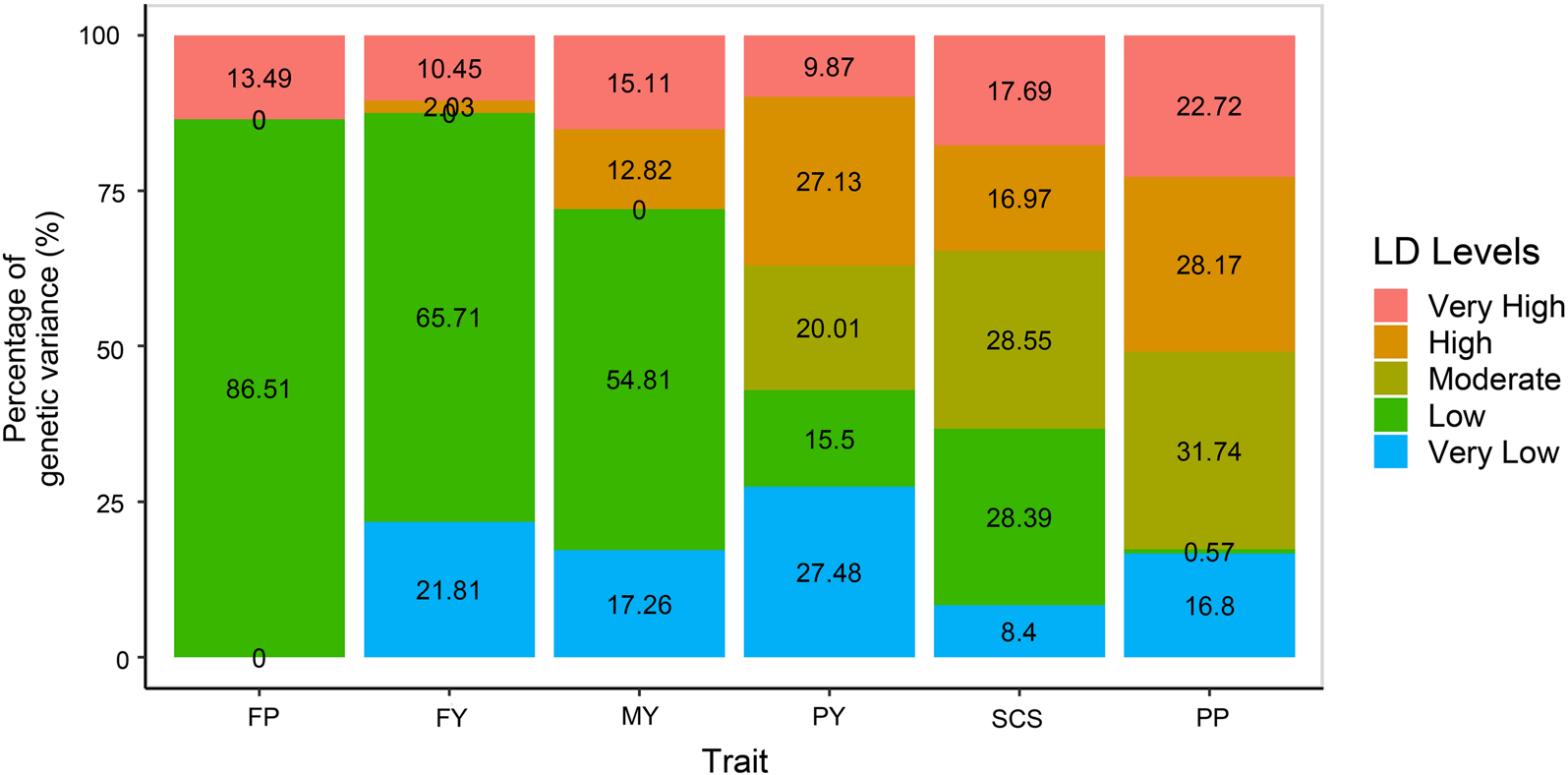
Percentage of genetic variance captured by SNPs at each LD level for dairy cattle traits. The GREML-LDS method was used to estimate the genetic variance of each LD level. SNPs in the high-density panel were used to construct the GRM used in GREML-LDS. The percentage of genetic variance captured by each LD level is the proportion of the genetic variance explained by that LD level to the genetic variance explained by all LD levels

Additional file 2: Fig. S3 shows the heritability enrichment of simulated phenotypes for each LD level. The estimates of heritability enrichment from GREML-LDS are consistent with the true value, which means that it is a reliable method to estimate the genetic contribution of SNPs at each LD level. Regardless of the genetic variance not captured by the 300K SNP data, GREML-LDS was used to estimate the contribution of SNPs at each LD level to the genetic variance of dairy cattle traits (Fig. 4). For the FP, FY and MY traits, the SNPs that contribute most of the genetic variance (72.1 to 86.5%) are those in LD levels 4 and 5, which means that most of the causal variants are located in regions of the genome of relatively low LD. For PY and SCS, the heritability estimates were almost evenly distributed among all the LD levels. For PP, SNPs in LD levels 1 and 2 contributed 50.9% of the genetic variance, which indicates that most of the causal variants are located in regions of the genome of relatively high LD. Thus, the causal variants of these quantitative traits were not evenly distributed among LD levels, and LD heterogeneity among regions should be considered when conducting genomic prediction and heritability estimation.

**Fig. 5:**
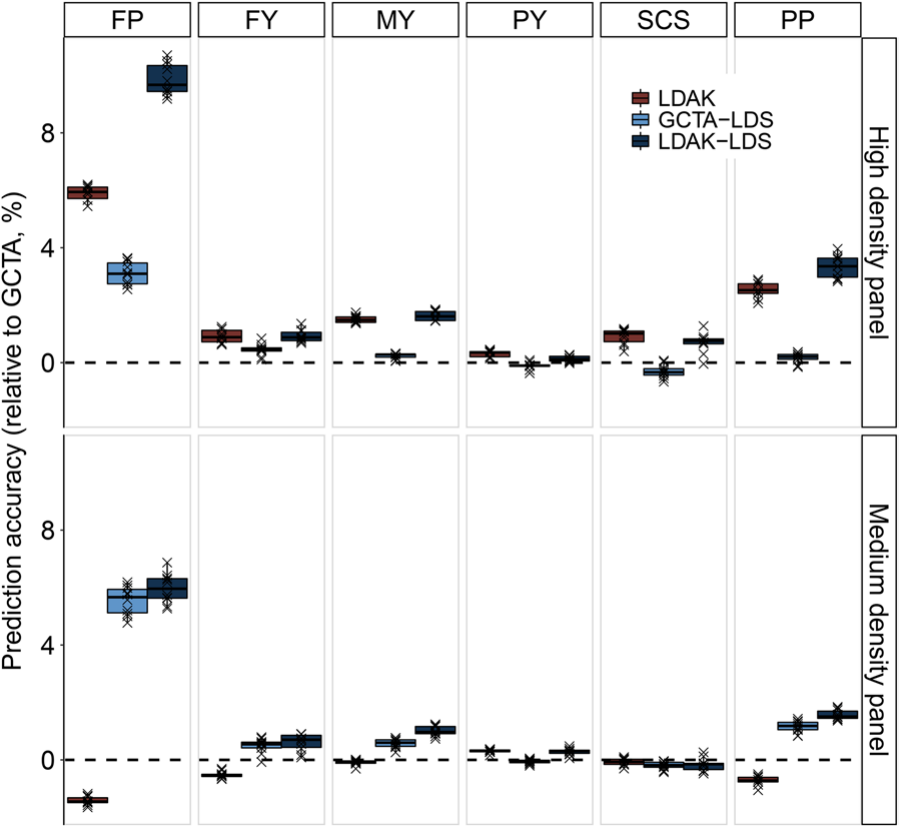
Genomic prediction accuracy of LDAK, GCTA-LDS and LDAK-LDS for dairy cattle traits based on high- and medium-density panels. The prediction accuracy was expressed as the percentage of increase in prediction accuracy relative to that obtained with the GCTA model. The boxes and points report the percentage increase in prediction accuracy relative to that with GCTA

Figure 5 shows the genomic prediction accuracy of the models that control LD heterogeneity among regions compared to the GCTA model for dairy cattle traits. The results of Fig. 5 combined with those of Fig. 4 show that more the distribution of the causal variants is uneven at each LD level, more does the advantage of the models that control LD heterogeneity among regions become obvious. This is consistent with the results of the simulation study (Fig. 2). When the high-density panel was used, the genomic prediction accuracy of LDAK, GCTA-LDS and LDAK-LDS was higher (0.3 to 10.7%) than that of GCTA for all traits (Fig. 5). When using the medium-density panel, both the advantage of the models that control LD heterogeneity among regions and the improvement in genomic prediction accuracy (− 0.1 to 6.9%) decrease. Similarly, for real traits, the higher is the genomic prediction accuracy of the model (see Additional file 1: Table S2), the better is the model fit (see Additional file 1: Table S3).

## Discussion

Currently, studies on LD heterogeneity among regions have focused on the estimation of the heritability of human complex traits and diseases [9, 10]. However, how does LD heterogeneity among regions affect genomic prediction and heritability estimation of livestock quantitative traits, and how can its adverse effects be eliminated are rarely investigated in the literature. In this study, we used LDAK, GCTA-LDS and LDAK-LDS to control LD heterogeneity among regions, and tested their effectiveness for genomic prediction and heritability estimation in dairy cattle. We found that GCTA-LDS and LDAK-LDS can effectively eliminate the adverse effects of LD heterogeneity among regions, and improve the accuracy of genomic prediction and unbiasedness of heritability estimates. Furthermore, the models that control LD heterogeneity among regions are more effective with high-density SNP data.

### Controlling LD heterogeneity among regions improves genomic prediction and heritability estimation

In this study, all GRM were constructed based on the method proposed by Yang et al. [23]. This method assumes that all causal variants contribute equally to heritability. The contribution of a causal variant to heritability can be divided into two parts: the genotype variance ($2{p}_{i}(1-{p}_{i}))$ and the effect variance (${a}_{i}^{2}$). The equal contribution of all causal variants to heritability means that ${h}^{2}=2{p}_{i}(1-{p}_{i}){a}_{i}^{2}$ of all causal variants are equal. In Eq. (2), all genotypes of each locus were standardized first, and then $\mathbf{XX}^{\prime}/N$ was used to construct GRM, which ensured the assumption that each locus contributed equally to heritability [23]. In this study, the simulation of the effect of the causal variant is consistent with this assumption (see the Methods section). Therefore, in the simulation study, if there are no other influencing factors apart from the allele frequency and the effect of the causal variant, the GCTA model constructed by the method of Yang et al. [23] should be unbiased for heritability estimation. As shown by Fig. 2b, when the causal variants of simulated phenotypes had an average tagging level, i.e., the causal variants were not affected by LD heterogeneity among regions, the heritability estimates based on the Yang et al.’s GRM were basically unbiased. However, when the causal variants of simulated phenotypes were in either the high LD regions (strongly tagging level) or low LD regions (weakly tagging level), i.e., the causal variants were affected by LD heterogeneity among regions, the estimates of heritability obtained with the GCTA model were not unbiased (Fig. 2b).

Due to LD heterogeneity among regions, the genetic contribution of causal variants was overestimated in high LD regions and underestimated in low LD regions [9, 29, 30]. Using LDAK to construct the LD-weighted GRM can increase the weight of weakly tagged SNPs [9], which is beneficial to genomic prediction and heritability estimation for traits that are mainly controlled by weakly tagged causal variants (Fig. 2a, b). LDAK can reduce the weight of strongly tagged SNPs [9], which is unfavorable for genomic prediction and heritability estimation of traits that are mainly controlled by strongly tagged causal variants (Fig. 2a, b). Previous studies have confirmed that increasing the weight of markers near the causal variants is beneficial for genomic prediction [22, 24, 31]. For the simulated phenotypes in this study, all the causal variants had the same genetic variance, which is consistent with the assumption of GCTA [23]. Therefore, in the simulation study, MAF does not affect the genetic variance, and LD heterogeneity among regions has to be accounted for in the construction of the GCTA-LDS and LDAK-LDS models [10]. Compared with GCTA and LDAK, GCTA-LDS and LDAK-LDS can improve the accuracy of genomic prediction and the unbiasedness of heritability estimates, regardless of the genetic architecture of the trait (Fig. 2a, b).

This result confirms that GREML-LDS is effective for heritability estimation and proves that GREML-LDS is also beneficial to genomic prediction for livestock populations. Thus, GCTA-LDS and LDAK-LDS can be used as reliable models to control LD heterogeneity among regions, and the LD stratified genomic best linear unbiased prediction (GBLUP) or the single-step GBLUP models can be constructed based on these methods to eliminate the adverse effects of LD heterogeneity among regions and improve the accuracy of genomic prediction in livestock.

It is generally assumed that the greater is the genetic variance (or heritability) explained by the model, the higher is the genomic prediction accuracy [20]. However, in the literature, there are exceptions to this relationship and for example, Ren et al. [18] reported estimates of heritability that increased while the genomic prediction accuracies remained unchanged or even decreased. By performing a joint analysis of the performance of each model in terms of genome prediction and heritability estimation, we found that more the heritability estimates were unbiased, the higher was the genomic prediction accuracy (Figs. 2 and 3). Therefore, it is difficult to judge the prediction performance of the model based on the heritability estimate, because in reality, the true heritability is difficult to obtain. At the same time, we found that model fit can be a reliable indicator of model performance in genomic prediction and heritability estimation. That is, the better is the performance of the model for genomic prediction and heritability estimation, the better is the model fit (Figs. 2 and 3). In fact, studies based on the heritability model usually select models based on model fit [32].

### Models that control LD heterogeneity among regions are more efficient with high-density data

Due to LD, the level of replicate tagging of SNPs is unevenly distributed along the genome (Fig. 1b) and (see Additional file 2: Fig. S1). SNPs in low LD regions have a lower level of replicate tagging and those in high LD regions have a higher level of replicate tagging (Table 1).

With the higher-density panel, the differences in the level of replicate tagging between SNPs in high and low LD regions increase (Table 1). Thus, increased marker density leads to underestimation of the genetic variance of the causal variants in low-LD regions, which results in reduced genomic prediction accuracy (Fig. 3a) and heritability estimates (Fig. 3b). This may be the reason why previous studies found that genomic prediction accuracy and heritability estimates decreased as marker density increased [18, 19]. Similarly, the relative weights of SNPs in high LD regions of the high-density panel are larger than those of the medium-density panel. For phenotypes that are mainly controlled by strongly tagged causal variants, the genetic contribution of the causal variants in the high-density panel was overestimated compared to that in the medium-density panel (Fig. 3e). Therefore, LD heterogeneity among regions is more obvious in high-density SNP data and has a greater impact on genome prediction and heritability estimation based on a high-density panel. Thus, to efficiently use high-density SNP data for genomic prediction or heritability estimation, LD heterogeneity among regions needs to be controlled. The use of GCTA-LDS or LDAK-LDS to control LD heterogeneity among regions can greatly improve the efficiency of high-density SNP data in genomic prediction and heritability estimation (Fig. 3). In contrast, controlling for the adverse effects of LD heterogeneity among regions with medium-density SNP data results in less improvement in genomic prediction and heritability estimation (Fig. 3). Therefore, models that control LD heterogeneity among regions are more efficient with high-density SNP data, which can be used to effectively exploit the potential of high-density SNP data in genomic prediction and heritability estimation.

### Controlling LD heterogeneity among regions improves genomic prediction accuracy for dairy cattle traits

The results of the estimation of variance components based on GREML-LDS showed that the causal variants of quantitative traits are not evenly distributed among LD levels (Fig. 4). As discussed above, with an increase in marker density, the genetic variance of the causal variants in the low LD regions will be underestimated and that of causal variants in the high LD regions will be overestimated, resulting in a decrease in genomic prediction accuracy of the models that do not control LD heterogeneity among regions. Compared with the medium-density panel, the genomic prediction accuracies obtained with the high-density panel decreased by − 0.4 to 5.8%, and the heritability estimates decreased by − 1.3 to 3.8% when using GCTA (see Additional file 1: Tables S2 and S4). Compared with the simulated traits (Fig. 3a–c), the prediction accuracies and heritability estimates of some of the real traits decreased more severely, which may be due to the complex genetic architecture of real traits. This is in agreement with results in the literature [18, 30], which suggest that the use of classical methods that do not take LD heterogeneity among regions into account will lead to an increased bias in heritability estimates as marker density increases. Therefore, classical models are not appropriate when using whole-genome sequence data or high-density SNP data.

In contrast to the simulated traits, LDAK-LDS gave the best genomic prediction results for almost all of the real dairy traits. In this case, SNPs should not only be grouped according to LD heterogeneity among regions but the SNPs in low LD regions should have a greater weight. Generally, SNPs in low LD regions also have a low MAF, and this is also true in the bovine genome data (see Additional file 2: Fig. S4). This means that loci with a low MAF need a greater weight, which may be related to negative selection, i.e. the lower the MAF, the larger the effect of the SNP. Previous studies have found that negative selection occurs frequently for human [12, 32–34] and cattle [35] complex traits. LDAK increased the weights of SNPs in the low LD region and also increased the weights of SNPs with a low MAF, which may be the reason for the outstanding performance of LDAK-LDS on real traits. Although LDAK and GREML-LDS were originally proposed to improve the unbiasedness of heritability estimates [9, 10], these models seem to be more effective for genomic prediction. For example, controlling for LD heterogeneity among bovine genomic regions, increased genomic prediction accuracy based on high-density SNP data by 0.3 to 10.7% (Fig. 5), while heritability estimates ranged from − 1.67 to 5.01% (see Additional file 1: Table S4), with a larger variation in genomic prediction accuracy than in heritability estimates. This may be because, in the GCTA model, the genetic variance in low LD regions that is underestimated, is compensated for by the genetic variance in high LD that is overestimated, which results in a smaller difference between the heritability estimates obtained with the models that control LD heterogeneity among regions and the estimates obtained with the GCTA model. Previous studies have found similar results [9]. For genome prediction, it is more important to accurately assess the genetic contribution of each genomic region than to estimate the total heritability. Models that control LD heterogeneity among regions can avoid underestimation of the genetic contribution of low LD regions and overestimation of that of high LD regions, which is very important for genome prediction.

Controlling for LD heterogeneity among regions significantly improved the accuracy of genomic prediction based on the high-density panel (Fig. 5), but it did not improve much compared to that obtained with the medium-density panel (see Additional file 1: Table S2). Therefore, for real traits, in addition to LD heterogeneity among regions, other key factors that affect the accuracy of genomic prediction and the unbiasedness of heritability estimates should be investigated. For example, the contribution of rare causal variants to genetic variance [12, 36], the presence of major genes [37], the distribution of marker effects [38] and the application of functional annotation in genomic prediction [39]. In addition, the combination of the effective methods to control LD heterogeneity investigated in this study, with trait-specific weighting methods [31], is expected to further improve the accuracy of genomic prediction and unbiasedness of heritability estimation.

## Conclusions

LD heterogeneity among regions has an adverse effect on genomic prediction and heritability estimation. Dividing SNPs into multiple LD levels based on regional LD and constructing an LD-stratified multi-component model can effectively eliminate the adverse effects of LD heterogeneity among regions and improve the accuracy of genomic prediction and the unbiasedness of heritability estimates. For WGS or high-density SNP data, the adverse effect of LD heterogeneity among regions is more obvious, and the LD-stratified multi-component model can greatly improve the efficiency of using high-density data in genomic prediction and heritability estimation. In addition, the model fit can be used as a reliable indicator to measure the performance of the model in genomic prediction and heritability estimation.

## Supplementary Information

**Additional file 1: Table S1.** Descriptive statistics of estimated breeding values and their reliabilities. **Table S2.** Genomic prediction accuracy of GCTA, LDAK, GCTA-LDS and LDAK-LDS based on the high- and medium-density panels for dairy cattle traits. **Table S3.** Model fit (AIC) of GCTA, LDAK, GCTA-LDS and LDAK-LDS based on the high- and medium-density panels for dairy cattle traits. **Table S4.** Estimates of SNP-heritability for dairy cattle traits by GCTA, LDAK, GCTA-LDS, and LDAK-LDS based on the high-and medium-density panels

**Additional file 2: Figure S1.** Replicate tagging of SNPs on chromosome 29 without and after LD weighting. (a) Represents replicate tagging of SNPs without LD weighting in the high-density panel (300K). (b) Represents replicate tagging of SNPs after LD weighting in the high-density panel. (c) Represents replicate tagging of SNPs without LD weighting in the medium-density panel (50K). (d) Represents replicate tagging of SNPs after LD weighting in the medium-density panel. (e) Represents the distribution of replicate tagging of SNPs without LD weighting in the high-density panel. (f) Represents the distribution of replicate tagging of SNPs after LD weighting in the high-density panel. (g) Represents the distribution of replicate tagging of SNPs without LD weighting in the medium-density panel. (h) Represents the distribution of replicate tagging of SNPs after LD weighting in the medium-density panel. **Figure S2.** Performance of GCTA, LDAK, GCTA-LDS and LDAK-LDS in terms of genomic prediction (a), heritability estimation (b), and model fit (c) for simulated phenotypes that are controlled by causal variants with different tagging levels. The heritability of all simulated phenotypes was 0.5. Paired *t*-test was applied to compare the difference between models, with *P* values adjusted by Bonferroni correction. ***indicates significant differences at *P* < 0.001, ** significant differences at 0.001 < *P* < 0.01, * significant differences at 0.01 < *P* < 0.05, and ns indicates no statistically significant difference. **Figure S3.** Estimated heritability enrichment of simulated phenotypes in five LD groups. Phenotypes in (a) to (e) were controlled by very weakly, weakly, averagely, strongly, and very strongly tagged causal variants, respectively. Estimates of heritability enrichment were calculated from the GREML-LDS model. SNPs in the high-density panel were used to construct the GRM used in GREML-LDS. The red lines represent the true median of heritability enrichment for simulated traits. **Figure S4.** Relationship between LD score and minor allele frequency. The 336,977 SNPs in the high-density panel were used to calculate the LD score. The red line is the regression line across all 336,977 SNPs, its positive gradient, is especially apparent for SNPs with a MAF < 0.2.

## Data Availability

Publicly available datasets were analyzed in this study. The SNP chip data and the EBV of the 2000 bulls are available at: https://www.g3journal.org/content/suppl/2015/02/09/g3.114.016261.DC1 (accessed on 5 February 2015). The phenotype simulation script and the regional LD calculation script used in this study are available at: https://github.com/SCAU-AnimalGenetics/LD-heterogeneity/tree/main/simphe and https://github.com/SCAU-AnimalGenetics/LD-heterogeneity/tree-save/main/regional-LD.
