# Supplementary material for: Impact of linkage disequilibrium heterogeneity along the genome on genomic prediction and heritability estimation
Source: Genet Sel Evol. 2022 Jun 27;54:47. doi: 10.1186/s12711-022-00737-3 (PMC9235212; doi:10.1186/s12711-022-00737-3)
Supplement: Supplementary file 1 — Additional file 1: Table S1. Descriptive statistics of estimated breeding values and their reliabilities. Table S2. Genomic prediction accuracy of GCTA, LDAK, GCTA-LDS and LDAK-LDS based on the high- and medium-density panels for dairy cattle traits. Table S3. Model fit (AIC) of GCTA, LDAK, GCTA-LDS and LDAK-LDS based on the high- and medium-density panels for dairy cattle traits. Table S4. Estimates of SNP-heritability for dairy cattle traits by GCTA, LDAK, GCTA-LDS, and LDAK-LDS based on the high-and medium-density panels [file 12711_2022_737_MOESM1_ESM.docx]

Table S1 The descriptive statistics of estimated breeding values and their reliabilities

| Traits | Estimated breeding values | | | | Reliabilities of estimated breeding values | | | |
| --- | --- | --- | --- | --- | --- | --- | --- | --- |
|  | Mean | Minimum value | Maximum value | Standard deviation | Mean | Minimum value | Maximum value | Standard deviation |
| Milk yield (MY) | 231.700 | -1800.000 | 2457.000 | 649.759 | 0.973 | 0.950 | 0.990 | 0.016 |
| Milk protein yield (PY) | 6.529 | -57.000 | 64.000 | 18.949 | 0.971 | 0.940 | 0.990 | 0.017 |
| Milk fat yield (FY) | 5.835 | -69.000 | 101.000 | 23.356 | 0.972 | 0.940 | 0.990 | 0.017 |
| Milk protein percentage (PP) | -0.010 | -0.430 | 0.540 | 0.119 | 0.971 | 0.940 | 0.990 | 0.017 |
| Milk fat percentage (FP) | -0.027 | -1.060 | 1.150 | 0.294 | 0.972 | 0.940 | 0.990 | 0.017 |
| Somatic cell score (SCS) | 103.100 | 58.000 | 140.000 | 11.587 | 0.942 | 0.890 | 0.990 | 0.039 |

Table S2 Genomic prediction accuracy of GCTA, LDAK, GCTA-LDS and LDAK-LDS based on high-density panel or medium-density panel for dairy cattle traits

| Traits | High-density panel (300 K SNPs) | | | | Medium-density panel (50 K SNPs) | | | |
| --- | --- | --- | --- | --- | --- | --- | --- | --- |
|  | GCTA | LDAK | GCTA-LDS | LDAK-LDS | GCTA | LDAK | GCTA-LDS | LDAK-LDS |
| FY | 0.666 | 0.672 | 0.669 | 0.672 | 0.677 | 0.674 | 0.680 | 0.681 |
| FP | 0.696 | 0.737 | 0.718 | 0.765 | 0.736 | 0.725 | 0.776 | 0.780 |
| MY | 0.711 | 0.722 | 0.713 | 0.723 | 0.720 | 0.719 | 0.724 | 0.727 |
| PY | 0.720 | 0.722 | 0.719 | 0.721 | 0.717 | 0.719 | 0.717 | 0.719 |
| PP | 0.661 | 0.678 | 0.662 | 0.683 | 0.669 | 0.664 | 0.677 | 0.679 |
| SCS | 0.618 | 0.623 | 0.616 | 0.622 | 0.616 | 0.616 | 0.615 | 0.615 |

Table S3 Model fit (AIC) of GCTA, LDAK, GCTA-LDS and LDAK-LDS based on high-density panel or medium-density panel for dairy cattle traits

| Traits | High-density panel (300 K SNPs) | | | | Medium-density panel (50 K SNPs) | | | |
| --- | --- | --- | --- | --- | --- | --- | --- | --- |
|  | GCTA | LDAK | GCTA-LDS | LDAK-LDS | GCTA | LDAK | GCTA-LDS | LDAK-LDS |
| FY | 4441.612 | 4418.727 | 4435.219 | 4413.402 | 4407.005 | 4416.695 | 4392.383 | 4389.943 |
| FP | 4396.793 | 4263.207 | 4304.951 | 4102.527 | 4253.627 | 4302.749 | 4019.261 | 4008.558 |
| MY | 4203.161 | 4163.277 | 4199.907 | 4156.282 | 4167.984 | 4176.600 | 4153.910 | 4148.514 |
| PY | 4143.037 | 4128.046 | 4144.381 | 4129.973 | 4145.748 | 4141.629 | 4148.617 | 4144.563 |
| PP | 4505.634 | 4455.382 | 4500.126 | 4436.445 | 4478.821 | 4486.407 | 4457.845 | 4447.227 |
| SCS | 4565.365 | 4551.703 | 4568.426 | 4549.776 | 4565.002 | 4564.231 | 4567.703 | 4564.277 |

Table S4 Estimates of SNP-heritability for dairy cattle traits by GCTA, LDAK, GCTA-LDS, and LDAK-LDS based on high-density panel or medium-density panel

| Traits | High-density panel (300 K SNPs) | | | | Medium-density panel (50 K SNPs) | | | |
| --- | --- | --- | --- | --- | --- | --- | --- | --- |
|  | GCTA | LDAK | GCTA-LDS | LDAK-LDS | GCTA | LDAK | GCTA-LDS | LDAK-LDS |
| FY | 0.837 | 0.851 | 0.838 | 0.844 | 0.850 | 0.852 | 0.841 | 0.843 |
| FP | 0.898 | 0.943 | 0.866 | 0.905 | 0.932 | 0.931 | 0.884 | 0.889 |
| MY | 0.814 | 0.839 | 0.814 | 0.830 | 0.824 | 0.832 | 0.818 | 0.825 |
| PY | 0.789 | 0.802 | 0.798 | 0.808 | 0.779 | 0.791 | 0.787 | 0.795 |
| PP | 0.896 | 0.920 | 0.885 | 0.906 | 0.906 | 0.906 | 0.894 | 0.896 |
| SCS | 0.754 | 0.780 | 0.749 | 0.772 | 0.754 | 0.763 | 0.748 | 0.756 |
