# Supplementary material for: Impact of linkage disequilibrium heterogeneity along the genome on genomic prediction and heritability estimation
Source: Genet Sel Evol. 2022 Jun 27;54:47. doi: 10.1186/s12711-022-00737-3 (PMC9235212; doi:10.1186/s12711-022-00737-3)
Supplement: Supplementary file 2 — Additional file 2: Figure S1. Replicate tagging of SNPs on chromosome 29 without and after LD weighting. (a) Represents replicate tagging of SNPs without LD weighting in the high-density panel (300K). (b) Represents replicate tagging of SNPs after LD weighting in the high-density panel. (c) Represents replicate tagging of SNPs without LD weighting in the medium-density panel (50K). (d) Represents replicate tagging of SNPs after LD weighting in the medium-density panel. (e) Represents the distribution of replicate tagging of SNPs without LD weighting in the high-density panel. (f) Represents the distribution of replicate tagging of SNPs after LD weighting in the high-density panel. (g) Represents the distribution of replicate tagging of SNPs without LD weighting in the medium-density panel. (h) Represents the distribution of replicate tagging of SNPs after LD weighting in the medium-density panel. Figure S2. Performance of GCTA, LDAK, GCTA-LDS and LDAK-LDS in terms of genomic prediction (a), heritability estimation (b), and model fit (c) for simulated phenotypes that are controlled by causal variants with different tagging levels. The heritability of all simulated phenotypes was 0.5. Paired t-test was applied to compare the difference between models, with P values adjusted by Bonferroni correction. ***indicates significant differences at P < 0.001, ** significant differences at 0.001 < P < 0.01, * significant differences at 0.01 < P < 0.05, and ns indicates no statistically significant difference. Figure S3. Estimated heritability enrichment of simulated phenotypes in five LD groups. Phenotypes in (a) to (e) were controlled by very weakly, weakly, averagely, strongly, and very strongly tagged causal variants, respectively. Estimates of heritability enrichment were calculated from the GREML-LDS model. SNPs in the high-density panel were used to construct the GRM used in GREML-LDS. The red lines represent the true median of heritability enrichment for simulated traits. [file 12711_2022_737_MOESM2_ESM.docx]

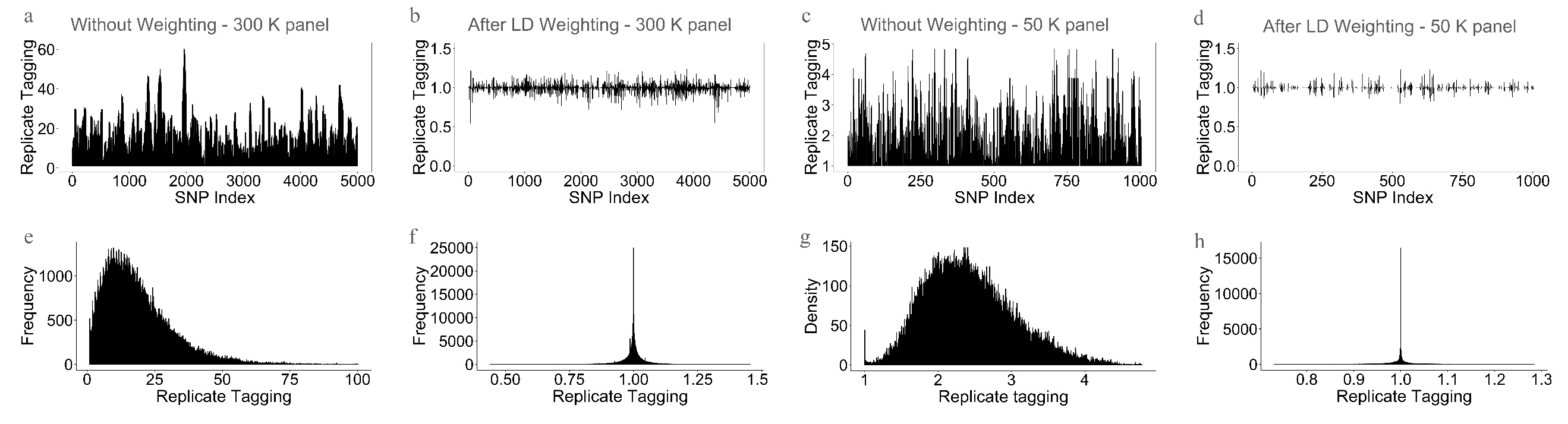


Figure S1 Replicate Tagging of SNPs on chromosome 29 without and after LD weighting. (a) represents replicate tagging of SNPs without LD weighting in the high-density panel (300 K). (b) represents replicate tagging of SNPs after LD weighting in the high-density panel. (c) represents replicate tagging of SNPs without LD weighting in the medium-density panel (50 K). (d) represents replicate tagging of SNPs after LD weighting in the medium-density panel. (e) represents the distribution of replicate tagging of SNPs without LD weighting in the high-density panel. (f) represents the distribution of replicate tagging of SNPs after LD weighting in the high-density panel. (g) represents the distribution of replicate tagging of SNPs without LD weighting in the medium-density panel. (h) represents the distribution of replicate tagging of SNPs after LD weighting in the medium-density panel.


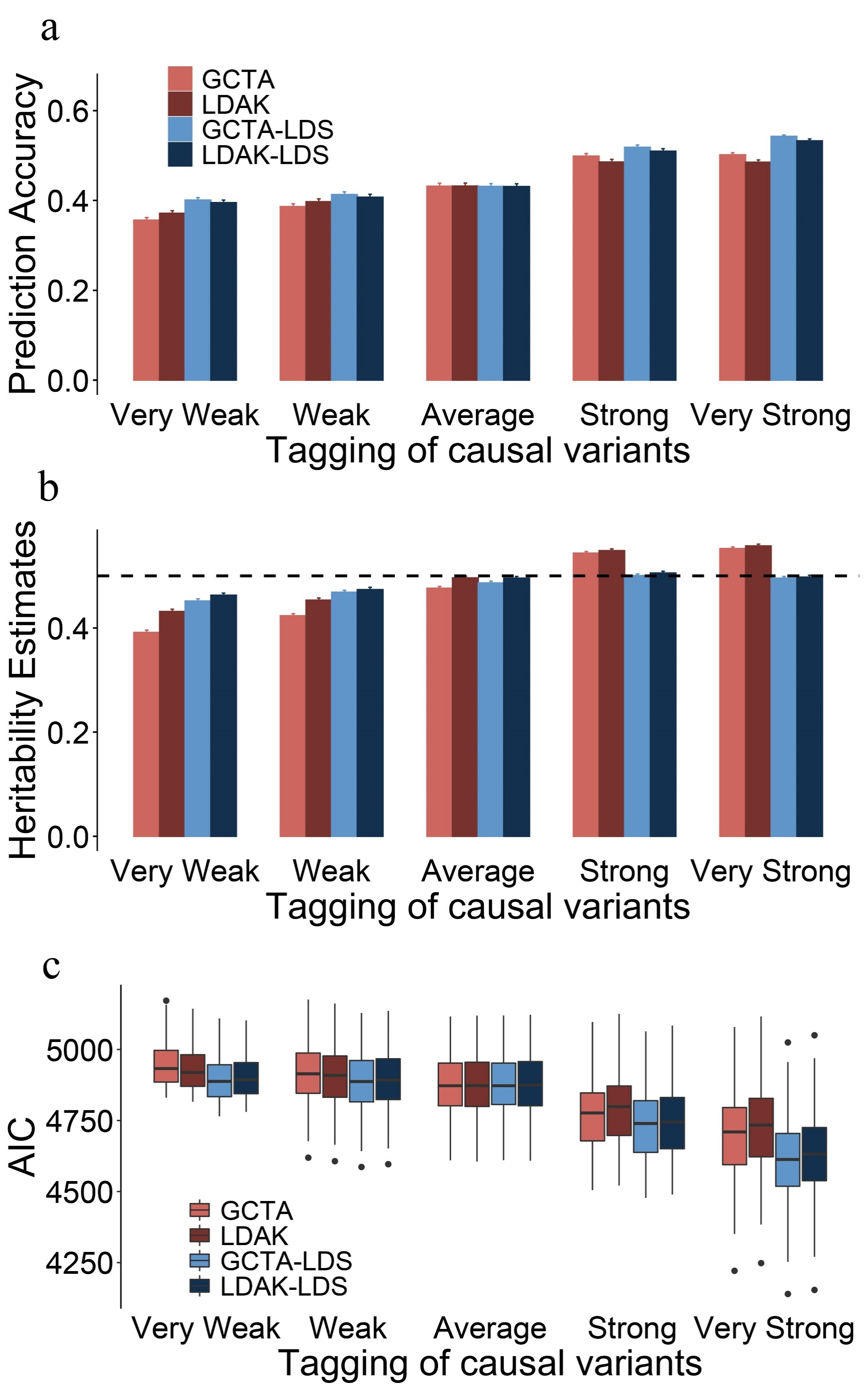


Figure S2 Performance of GCTA, LDAK, GCTA-LDS and LDAK-LDS in genomic prediction (a), heritability estimation (b), and model fit (c) for simulated phenotypes controlled by causal variants with different tagging levels. The heritability of all simulated phenotypes was 0.5. Paired *t*-test was applied to compare the difference between models, with *P* values adjusted by Bonferroni correction. ***indicates significant differences at *P* < 0.001, **indicates significant differences at 0.001 < *P* < 0.01, *indicates significant differences at 0.01 < *P* < 0.05, ns indicates no statistically significant difference.


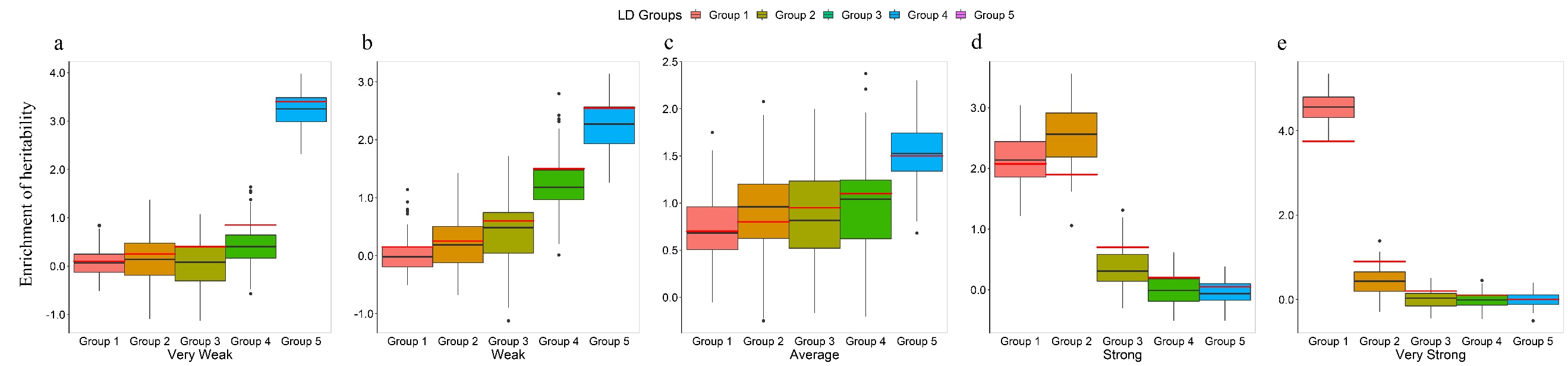


Figure S3 Estimated heritability enrichment of simulated phenotypes in five LD groups. Phenotypes in a-e were controlled by very weakly, weakly, averagely, strongly, and very strongly tagged causal variants, respectively. Estimates of heritability enrichment were calculated from the GREML-LDS model. SNPs in the high-density panel were used to construct the GRMs used in GREML-LDS. The red lines represent the true median of heritability enrichment for simulated traits.


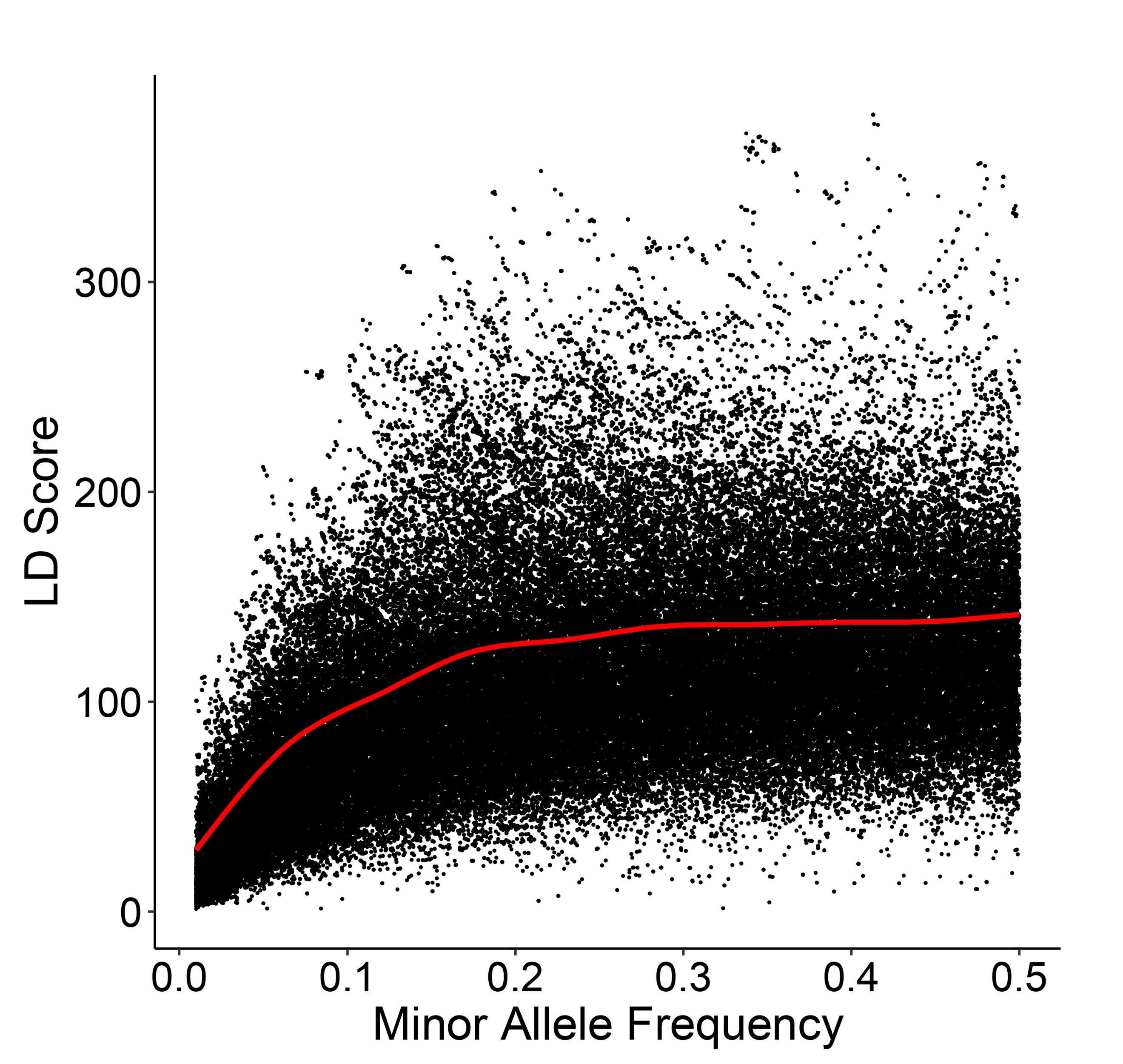


Figure S4 Relationship between LD score and minor allele frequency. The 336, 977 SNPs in the high-density panel were used to calculate LD Score. The red line is the regression line across all 336, 977 SNPs, its positive gradient, especially apparent for SNPs with MAF <0.2.
